# The effect of Artificial Intelligence Health Education Accurately Linking System on childhood asthma: study protocol for a pilot randomized controlled trial

**DOI:** 10.3389/fpsyt.2025.1585702

**Published:** 2025-06-06

**Authors:** Huan-Fang Wang, Yun-Hua Li, Qiaoling Zhang, Lihong Han, Lirong Wang, Lifang Zhang, Xue Bai, Mingyue Cheng, Ting Zhang, Fang Zhao, Hui Li, Xiao-Yun Wang

**Affiliations:** ^1^ Nursing Department, Inner Mongolia Maternal and Child Health Care Hospital, Hohhot, China; ^2^ College of Education, Chengdu College of Arts and Sciences, Chengdu, China; ^3^ Pediatric Internal Medicine Department, Inner Mongolia Maternal and Child Health Care Hospital, Hohhot, China; ^4^ Pediatric Outpatient Department, Inner Mongolia Maternal and Child Health Care Hospital, Hohhot, China; ^5^ Nursing Department, Shandong Provincial Hospital Affiliated to Shandong First Medical University, Jinan, China; ^6^ Pediatric Department, Inner Mongolia Maternal and Child Health Care Hospital, Hohhot, China

**Keywords:** artificial intelligence, large language model, childhood asthma, chronic disease, mobile health, RCT

## Abstract

**Background:**

Childhood asthma is a prevalent chronic disease that affects millions of children worldwide. Managing this disease demands not only medical treatment but also the long-term self-management efforts of both children and their parents. Conventional self-management education typically depends on face-to-face approaches, often failing to take into account the personalized requirements and ongoing support needed. Nevertheless, with the evolution of artificial intelligence (AI) technology, fresh prospects have emerged to boost the effectiveness of self-management for childhood asthma. Based on it, we have designed an AI Health Education Accurately Linkage System (AI-HEALS) to explore whether AI-driven interventions can improve self-management capabilities of families with asthmatic children, thereby helping them control the disease and reduce medical costs.

**Methods:**

This research is a pilot single-blind randomized controlled trial (RCT) intended to gauge the efficacy of the AI-HEALS intervention delivered via the WeChat platform in enhancing the self-management abilities of families with asthmatic children. Participants will be recruited from eligible families whose children have been diagnosed with asthma and randomly allocated to either the intervention group or the control group. The control group will receive standard treatment, whereas the intervention group will receive both standard treatment and the AI-HEALS intervention. The intervention includes an AI-enabled, voice-activated interactive question-and-answer system, as well as monitoring and recording of physiological indicators, regular reminders, and customized educational articles. All components of the intervention will mainly be provided through a WeChat official account named “Children’s Asthma Health Management Expert.” AI-HEALS will construct its knowledge base according to pediatric asthma treatment guidelines to enhance the accuracy and reliability of the information it offers. The primary outcome measure is the alteration in asthma symptom control levels, while secondary outcomes comprise a variety of other physiological indicators related to asthma, parents’ self-management behaviors, and mental health conditions.

**Discussion:**

This study combines AI and mobile health technology to develop the AI-HEALS system, with the aim of assisting families of children with asthma in controlling the disease symptoms. The primary objective is to evaluate whether the intervention can improve asthma symptom control.

**Clinical trial registration:**

The study is scheduled to begin in April 2025 and is expected to conclude in December 2026. This research protocol is the first version and was registered with the China Clinical Trial Registration Center on February 14, 2025 (Registration Number: ChiCTR2500097233).

## 1 Introduction

Childhood asthma ranks among the most prevalent chronic diseases in children globally. A report from the World Health Organization indicates that around 260 million people worldwide are affected by asthma, with roughly 10% of these cases occurring in children (1, 2). The prevalence of asthma varies significantly across countries, exhibiting distinct trends and characteristics (2, 3). In the African region, current and lifetime asthma prevalence rates are the highest, at 13.2% and 11.3% respectively (2, 3). By contrast, the Americas have the lowest current asthma prevalence, which is 10.0%, while Southeast Asia reports the lowest lifetime asthma prevalence, at 8.8% (2, 3). Moreover, environmental factors associated with urbanization, lifestyle changes, as well as genetic factors, are all regarded as crucial factors contributing to childhood asthma development (4, 5).

Childhood asthma does not merely lead to recurrent wheezing, coughing, and breathing difficulties (6, 7). It can also trigger severe acute episodes that pose direct threats to the child’s life (6, 7). Additionally, asthma can have long-term implications for both the physical growth and mental health of children (8, 9). For example, school-aged children might have to miss school, leading to declining academic performance, and experience challenges with self-esteem and social skills due to their condition (8, 9). From a family perspective, the long-term management of asthma imposes a significant burden on daily life (10, 11). Families must continuously adapt their daily routines to meet the child’s medical requirements, like making frequent trips to the hospital and consistent medication administration (10, 11). Moreover, parents often endure heightened psychological stress and financial strain, especially in areas with scarce healthcare resources, where this burden is particularly pronounced (10, 11). Beyond the impact on individuals and families, childhood asthma also imposes substantial economic burdens on public health systems (12). One study estimates that over the next 20 years, asthma will incur approximately $300 billion in direct healthcare costs in the United States alone (13).

At present, the interventions for childhood asthma primarily comprise two major categories: pharmacological and non-pharmacological treatments. Pharmacological treatment relies on short-acting and long-acting bronchodilators, along with corticosteroids. These medications are critical for managing symptoms and preventing exacerbations (14, 15). In terms of non-pharmacological treatments, they include educational interventions, environmental control, and lifestyle adjustments. For instance, children are advised to avoid tobacco smoke and household allergens while increasing physical activity (14, 16). Although the existing intervention strategies demonstrate effectiveness, they face notable limitations and challenges. Firstly, pharmacological treatments may lead to dependency issues (17). Prolonged use may cause adverse effects, and their efficacy may vary among children (17). Secondly, non-pharmacological treatments require significant resources from both the family and the larger social environment. As a consequence, their implementation is not only difficult but also costly (6, 16). Additionally, current interventions are often not personalized, failing to fully account for individual circumstances and needs while inadequately integrating patient education and behavioral support (6). Given these limitations, the development of novel therapeutic strategies is urgent, particularly those leveraging digital health technologies and mobile health platforms.

Nowadays, more than 75% of the global population has access to the internet via mobile devices, and over 57% of households are connected to the internet. This clearly shows the wide-spread presence and importance of mobile internet in modern life, notably in health management and lifestyle adjustments. Research on the effectiveness of mobile health interventions and digital health interventions for patients has been conducted (18, 19). Mobile health applications have developed from simple voice and text message services to complex smart applications that incorporate various health behavior change programs (18–20). These interventions now address a broad range of conditions, including pain management, chronic disease management, weight control, and smoking cessation (18, 19). In recent years, mobile health interventions in asthma management have emerged as promising tools. For example, a randomized trial of the AIM2ACT program conducted by Fedele et al. demonstrated clinically significant improvements in asthma control among adolescent patients, accompanied by high participant satisfaction and engagement rates (21). Similarly, an evaluation of the “Breath Kit” mobile application led by Smith et al. reported significant improvements in inhaler technique adherence, with high user satisfaction and preference for digital delivery over traditional methods (22). Furthermore, a longitudinal study of the MASK-air^®^ application conducted by Kvedarienė et al. indicated high satisfaction levels and symptom improvements, with many patients recommending the app to others (23).

The Artificial Intelligence Health Education Precision Accurately System (AI-HEALS) is implemented via WeChat, China’s largest social platform with over 1.2 billion monthly active users. This platform can leverages WeChat’s ecosystem integration (Mini Programs, official accounts, payment systems) to embed health services into daily user behavior, overcoming retention challenges of standalone apps. Its demographic reach across urban/rural areas and older adults ensures equitable access, while closed-group chat and privacy features address health data sensitivity. Compared to platforms like Weibo (public content focus), WeChat’s social communication capabilities foster sustained peer support critical for behavior change. Based on it, this system utilizes low-cost, widely accessible mobile communication technology to deliver convenient and continuous disease management support directly to patients’ homes. AI-HEALS is grounded in a comprehensive theoretical framework that integrates the Multi-Theory Model (MTM) of health behavior change with the Health Action Process Approach (HAPA), forming the HAPA-MTM model (24, 25). This model is composed of three key phases: the pre-intentional phase, providing a comprehensive and continuous framework for health promotion. Interventions based on this model feature core components including: an intelligent question-and-answer system with voice interaction capabilities built on a proprietary knowledge base, monitoring of physiological parameters and behavioral patterns, customizable reminder systems, and personalized health content delivery. This innovative intervention framework is expected to better meet modern health management demands while providing evidence-based solutions for childhood asthma patients and their families.

In summary, this study aims to assess the effectiveness of the AI-HEALS intervention program for childhood asthma management through a pilot randomized controlled trial (RCT) across the following dimensions: asthma control, quality of life, medication adherence, and mental health outcomes.

1. Assess the extent to which a three-month intervention program grounded in the AI-HEALS improves clinical outcomes in childhood asthma patients’ physiological indicators.
2. Evaluate how effectively the three-month comprehensive intervention program can improve the psychological indicators of the parents of children with asthma.
3. Ascertain the effectiveness of the three-month comprehensive intervention program in promoting self-management behaviors among the parents of children with asthma.

## 2 Materials and methods

### 2.1 Design

**Figure 1:**
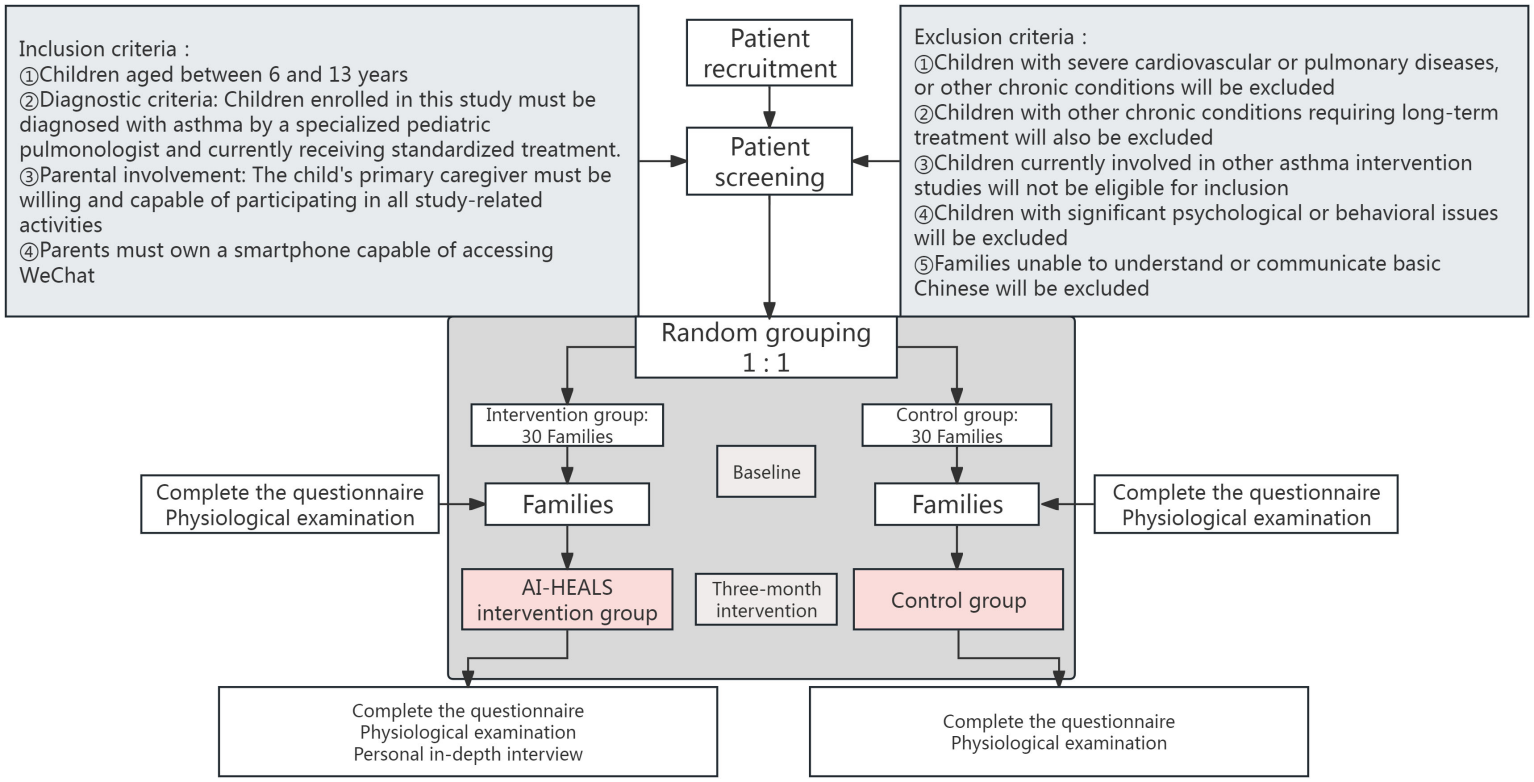
Flow chart of patient recruitment and study implementation.

This research is a pilot single-blind RCT. The participants are families of children who have been diagnosed with asthma at a specialized hospital located in the Inner Mongolia Autonomous Region of China. The study procedure and the method of grouping are presented in **Figure 1**.

### 2.2 Randomization and blinding

In this study, the randomization procedure uses a computer-generated sequence of random numbers. Patients will be assigned to the intervention and standard care groups in a 1:1 ratio. This randomization process will take place prior to patient admission. The aim is to ensure that each patient is assigned to the appropriate study group in a predefined order, thus ensuring randomization and fairness of allocation. When patients are admitted to the hospital, they will be assigned a unique code. This code is consistent with the sequence of random numbers and ensures smooth and consecutive enrollment of patients. Once randomization is complete, patients are directed to the appropriate hospital room based on their grouping. Specifically, patients in the intervention group are sent to Ward A, while those in the control group are sent to Ward B to prevent any possible cross-talk between the different interventions.

To ensure the confidentiality of the allocation sequence, an independent team member will be responsible for the randomization process and be neither involved in participant registration nor in intervening in the allocation. This is to prevent disclosure of the sequence during the course of the study. This team member then forward the allocation results to the intervention team. By doing this, both the participant enrollment team and the intervention team know nothing about the allocation in advance.

Finally, given the uniqueness of the AI-HEALS intervention in this trial, complete blinding of participants and therapists will be infeasible. This limitation may introduce biases such as performance bias due to participants’ awareness of their treatment allocation and detection bias from therapists’ knowledge of the implemented intervention. To minimize these biases, a series of measures will be implemented to reduce bias impact on this study. First, the identities of all intervention and control group participants will remain concealed until study completion. Second, an independent team member not involved in participant enrollment or intervention delivery will oversee the randomization process. This individual will directly forward allocation results to the intervention team to ensure both the enrollment and intervention teams remain unaware of group assignments in advance. Third, intervention group patients will be hospitalized in Ward A while controls occupy Ward B to prevent cross-contamination between groups. Finally, to further mitigate bias, research assistants blinded to group assignments will collect study data. Additionally, data analysts will remain blinded to group allocations during the analysis phase. Data will be coded and kept confidential throughout the study to preserve the integrity of the blinding process.

### 2.3 Study sample

This research will adopt a continuous enrollment method to recruit families of children diagnosed with asthma from April 2025 to December 2026. The detailed inclusion and exclusion criteria are as follows:

#### 2.3.1 Inclusion criteria

- Age range: The children should be between 6 and 13 years old.
- Diagnostic criteria: Children enrolled in this study must be diagnosed with asthma by a specialized pediatric pulmonologist and currently receiving standardized treatment. The diagnostic criteria are based on two components: 1) clinical manifestations including recurrent wheezing, cough, and shortness of breath (particularly exacerbated at night or post-exercise) (26); and 2) objective pulmonary function test results demonstrating reversible airflow limitation. Specifically, for children with recurrent cough and/or wheezing: forced expiratory volume in one second (FEV_1_) must be <80% of predicted value with an FEV_1_/forced vital capacity (FVC) ratio <0.8, and following inhalation of a short-acting β_2_-agonist, FEV_1_ increases by ≥12% and absolute improvement ≥200 mL (26). Children not meeting both diagnostic components will be excluded after physician confirmation.
- Parental involvement: The child’s primary caregiver must be willing and capable of participating in all study-related activities, including uploading daily life records (e.g., medication adherence logs, physical activity records), engaging in interactive health consultations with AI-HEALS, receiving and reviewing health education articles delivered by the system, and completing all study assessments.
- Technology requirements: The parents must possess a smartphone that can access WeChat and be willing to utilize the platform to receive and reply to the intervention content of the study.

#### 2.3.2 Exclusion criteria

- Severe comorbidities: Children diagnosed with specific severe cardiovascular diseases (e.g., congestive heart failure, uncontrolled arrhythmia, or myocardial infarction) or severe pulmonary diseases (e.g., cystic fibrosis) within the past six months will be excluded from this study. These conditions can significantly impair respiratory function and potentially influence intervention outcomes.
- Other chronic diseases: Children with other long-term chronic conditions that demand continuous treatment and could interfere with asthma management, like cystic, will also be ineligible.
- Participation in other asthma studies: Children who are currently taking part in other asthma intervention studies will not be included.
- Psychological or behavioral issues: Children with severe psychological or behavioral issues, such as schizophrenia, severe autism spectrum disorder, or other mental illnesses requiring acute hospitalization, will be excluded. These conditions can impede patients’ continuous participation in intervention activities and compromise the integrity of data collection.
- Language barriers: Families that are unable to understand or communicate in basic Chinese will not be part of the study.

#### 2.3.3 Withdrawal criteria

- Voluntary withdrawal: Families have the option to withdraw from the study for personal reasons. For example, they might face scheduling conflicts that prevent them from devoting the necessary time to the study, or their interest in participating may wane over time.
- Severe adverse events: Participants who encounter serious adverse events, such as severe physical illnesses or acute incidents like a heart attack or severe infection, which necessitate the interruption of the intervention, will be removed from the study.
- Changes in treatment: If a participant’s condition changes, or they receive medical advice to alter their treatment plan in a way that conflicts with the study intervention, they will be excluded.

### 2.4 Sample size

Given that this study is a pilot RCT, its primary focus is on evaluating the feasibility and practical implementation of the AI-HEALS intervention. Therefore, it is not designed to perform statistical hypothesis testing but rather to collect preliminary data on intervention operability and acceptability. As such, the sample size estimation for this pilot RCT does not follow traditional power analysis methods used for hypothesis testing. Instead, a pragmatic rule of thumb was applied to select a sample size sufficient to determine outcome variability and identify any operational issues (27). In line with the suggestions put forward by Billingham et al., the sample size for this pilot study is set at around 25 participants per group, amounting to a total of 50 participants (27). Anticipating an attrition rate of 20%, we determined that a minimum total sample of 60 participants (30 per group) would be necessary.

### 2.5 Recruitment

This study endeavors to recruit families of children with asthma who satisfy the inclusion and exclusion criteria at the target hospital. The recruitment process will be spearheaded by the hospital’s medical team. They will identify and extend invitations to eligible patients when they are admitted to the hospital. Subsequently, the research team will carry out an initial face-to-face communication with the patients. During this communication, detailed information about the study’s objectives, procedures, potential benefits, and possible risks will be provided. Eligible patients will be invited to the hospital for a thorough health assessment. This assessment includes a detailed review of the child’s asthma history and symptoms. After making certain that the patients have a full understanding of the study and answering any questions they might have, the families of children with asthma will be requested to sign an informed consent form. Only those who sign the informed consent form will be formally enrolled in the study.

Participants will receive a three-month AI-HEALS intervention. Additionally, this study employs fixed measurement agents to ensure reliability of self-report measures. Specifically, the same family member who initially accompanied the child to the first hospital visit will be designated to complete all self-report assessments throughout the study period to maintain measurement consistency. To encourage maximum participation, as an incentive for their involvement, all participants will receive a free medical consultation after the study.

### 2.6 Informed consent

The informed consent procedure will be conducted in an open, transparent manner and will strictly adhere to ethical standards. Parents or guardians of children meeting the inclusion and exclusion criteria will receive a comprehensive written explanation describing the research objectives, procedures, potential risks and benefits, as well as data privacy protection measures. This includes detailed information on how their data will be collected, stored, and protected. We will ensure that all data is securely stored in encrypted form on protected servers, accessible only to authorized research team members during the study period, and properly destroyed after the study concludes. Participants will be given sufficient time to carefully read the consent form, ask any questions, and provide consent voluntarily. They will be explicitly informed that they have the right to withdraw from the study at any time without any negative consequences. The research team will provide ongoing contact information for consultation, allowing participants to obtain further information or clarification when needed. Throughout the study, we will provide participants with any new information that might affect their decision to continue participation, ensuring continuous informed consent.

Additionally, participants will not face any penalties for deciding to withdraw from the intervention arm. If the intervention has already commenced, patients cannot switch to the control group after withdrawal and will be marked as an attrition case. Where feasible, semi-structured interviews will be conducted with attrited participants to explore reasons for discontinuation. During the informed consent process, participants will be explicitly informed of all available options following withdrawal, ensuring they remain fully aware of their rights and choices throughout the study period.

### 2.7 Interventions

#### 2.7.1 Control group: standard treatment group

Participants assigned to the control group will receive standard medical care delivered by a multidisciplinary clinical team. This care includes: 1) regular physician consultations for health assessment and treatment plan adjustments; 2) scheduled follow-up visits at predefined intervals to monitor disease progression; 3) comprehensive diagnostic evaluations to track health indicators; 4) structured health education sessions focusing on condition management, treatment adherence, and lifestyle modifications; and 5) evidence-based referrals to specialized care providers when clinically indicated. This integrated approach ensures alignment with clinical practice guidelines while maintaining ethical research standards and routine asthma management protocols.

#### 2.7.2 Intervention group: standard treatment + AI-HEALS

Building on previous pediatric asthma RCTs (28, 29), this study adopted a 3-month intervention duration. During the three-month period, the AI-HEALS intervention will be delivered to the intervention group via the ‘Children’s Asthma Health Management Expert’ WeChat platform. The intervention team comprises public health experts, clinicians, clinical nurses, psychologists, and statistical analysts, each contributing their unique expertise to the intervention design. The intervention program comprises the following four core components:

- Children’s Asthma Knowledge-Based Intelligent Question-Answering System (KBQA): This program aims to help parents of asthmatic children better understand asthma. It plays a role through the FAQ robot (FAQrobot), so that families can efficiently and accurately obtain the knowledge related to childhood asthma. In order to achieve this goal, we will develop a thorough and accurate knowledge base around childhood asthma. The knowledge base will cover a wide range of aspects, including the basic concepts of disease, etiology, symptoms, treatment methods, drug use, daily management, appropriate diet and appropriate sports activities. We will integrate this knowledge base into the large language model of Doubao (developed by ByteDance). In this way, the model can learn from the database and provide professional answers to patients’ questions directly on the WeChat platform. In addition, in order to enhance the interaction and user participation, the system will ask three related questions each time the user queries. This encourages users to explore further and interact more deeply with the system. In order to continuously optimize the knowledge base and better meet the needs of users, we will record the query and usage behavior of participants at the back end of the system, such as the time and frequency of accessing the system. By analyzing this data, we can identify the patterns of participants’ behavior. Based on these insights, we will be able to adjust and improve the knowledge base and question-and-answer system to ensure that the information is up-to-date and accurate. In the whole question-and-answer interaction process, the system supports text and voice input. This greatly reduces the threshold for users operating the system, so that more people can access the system.
- Recording and Monitoring of Physiological Indicators and Lifestyle Changes: In this study, the AI-HEALS will be used to encourage childhood asthma families to regularly record lifestyle factors, including weekly medication adherence, dietary habits, and physical exercise. Participants can directly input data into the system, allowing researchers to review recorded content in real-time through a backend interface, enabling long-term tracking and monitoring of self-management behaviors. To ensure data relevance and accuracy, the research team will jointly develop SMART (Specific, Measurable, Achievable, Relevant, Time-bound) plans with families during the initial phase of engagement. Data collection processes will be structured around these plans, with system-generated reminders sent every Saturday prompting detailed information uploads. This approach allows comprehensive assessment of intervention impacts on patients’ daily lives while ensuring data alignment with family-specific goals.
- Customized Reminder Services: In order to improve treatment compliance, the AI-HEALS system will provide a personalized reminder service. Participants can flexibly set reminders for various important tasks, such as medication and exercise. Once these reminders are set, the system will automatically send notifications at the specified time. This reminder service is designed to help patients strictly abide by medical advice, promote the long-term sustainability of healthy behaviors, and ultimately help to better control asthma.
- Automated and Personalized Asthma Science Articles for Family: The system will also run a mechanism to automatically distribute educational articles. Every week, families of children with asthma will automatically receive one to three articles on asthma management. These articles will cover a variety of topics. It includes dietary recommendations, such as which foods are good for asthmatic children and which foods should be avoided. Sports suggestions will also be provided to guide children to carry out sports activities of the appropriate type and intensity. It will also provide information on the correct medication, such as the correct method of medication and potential side effects that should be paid attention to. According to the interactions between the family and the system, such as the types of questions they raised, the frequency of visits, and the topics they are more interested in, targeted educational articles will be pushed accurately to meet the unique needs of each family. In order to maximize the effectiveness of information dissemination, the research team will regularly review the background data. This includes analyzing the number of articles that the participants visited, the different types of articles they liked, and the length of time they spent reading each article. According to this data, the article content and dissemination frequency will be adjusted.

The “Doubao-pro-32k” large language model (LLM) used in this study is a Chinese-trained Transformer-based architecture optimized for medical knowledge application. It was developed using a combination of biomedical literature and asthma-specific clinical guidelines, with fine-tuning conducted on a 32k context window to enable generation of long-form health education content. Prior to domain-specific fine-tuning, the model underwent extensive general language understanding pre-training to enhance its medical reasoning capabilities. Performance benchmarking was conducted using the third-party evaluation platform FlagEval (https://flageval.baai.ac.cn), where “Doubao-pro-32k” demonstrated competitive scores compared to GPT-4o. Specifically, Doubao-pro-32k attained an overall score of 77.75, surpassing GPT-4o’s 73.51. Its most distinguishing advantage lies in knowledge application (Doubao-pro-32k: 91.14 vs. GPT-4o: 86.71), a capability particularly crucial for generating evidence-based clinical information. Upon completion of development, an initial review of the model’s outputs was conducted by experts in the field to ensure that the information it generates matches well with existing medical knowledge and meets the high standards required for clinical research. In addition, at the end of the Pilot RCT, this study will set up a continuous validation process in which two experts will continuously review 10% of the outputs back to back on a monthly basis and calculate their correctness rate to maintain the accuracy of the system.

Finally, within the scope of this study, it is very important to protect the privacy of participants and ensure data security during their interaction with the AI-HEALS system. Any patient information obtained through the AI-HEALS platform will be treated with the strictest confidentiality and will only be used for research purposes. Each member of the research team is bound by the relevant privacy and confidentiality agreements. This means that they have a legal and moral obligation to protect the participants’ sensitive data. In addition, participants have the right to unconditionally withdraw from the study at any stage without providing any reason. This right respects the autonomy of participants and ensures that they will not be forced to continue participating in research. In addition, all adverse events related to the intervention will be recorded and reported in detail in strict accordance with local regulations and established procedures.

### 2.8 Strategies to improve adherence to interventions

To encourage participant adherence, we will (1) provide a comprehensive explanation of participants’ responsibilities during the observation period, both in the recruitment program and when signing the informed consent form. Simultaneously, we will work to establish strong relationships with participants, aiming to foster mutual trust. (2) Throughout the research, researchers will offer all-round education and guidance regarding disease management and preventive measures. This involves elaborating on the characteristics of the disease, underscoring the significance of strictly following the treatment plan, and sharing strategies for preventing potential complications. (3) On a weekly basis, the research team will assess the interaction between the intervention group and the system. This is to make sure that participants are sufficiently engaged. Regular monitoring allows for timely identification of any issues or disengagement, enabling the team to take corrective actions and keep participants involved.

### 2.9 Outcomes

#### 2.9.1 Primary outcomes

The primary outcome was changes in asthma symptom control, measured using the Childhood Asthma Control Test (C-ACT) at baseline, discharge, and post-intervention. Given the nested longitudinal structure of repeated measurements within families, we employed multilevel negative binomial models to analyze asthma symptom frequency (count data), estimating incidence rate ratios (IRRs) and 95% confidence intervals (CIs).

#### 2.9.2 Secondary outcomes

- Physiological Control of Children: The assessment indicators cover multiple aspects. In terms of pulmonary function, key parameters such as pulmonary ventilation function and expiratory flow rate will be detected by professional equipment; the average number of days with asthma symptoms, record the number of days when children experience asthma-related symptoms within a certain period; the number of nights awakened due to asthma symptoms; the number of days with activity limitations due to asthma; the number of days per week requiring inhaled bronchodilators for symptom relief; height, weight, waist circumference, body mass index (BMI); the 30 - day readmission rate; the length of hospital stay.
- Parental Behavior Changes: Parental behavioral changes will be measured using specific instruments, including a self-developed questionnaire to assess frequency and exposure to smoking and alcohol consumption; the Pittsburgh Sleep Quality Index Short Form (PSQI-SF) to evaluate sleep patterns; and the EQ-5D-5L (EuroQol-5 Dimensions 5 Levels) scale for measuring overall quality of life.
- Parental Social Cognition and Psychology: Self-efficacy; depression, anxiety, stress levels; social support.

In addition, the recruitment rate, dropout rate, and reasons for withdrawal will be collected. During the data analysis phase, in order to assess the homogeneity of the data, the demographic characteristics and study outcomes of those who dropped out will be compared to determine whether the dropout situation has an impact on the overall effectiveness of the study.

### 2.10 Variables measurement

At the baseline stage, sociodemographic variables will be collected. Physical examinations and questionnaires are scheduled to be completed at the baseline, upon discharge, and at the end of the intervention (**Table 1**).

- Sociodemographic Variables: A custom-designed demographic questionnaire will be utilized to measure the following variables: the child’s gender and age, the ages of the parents, marital status, type of household registration, number of persons in the household, education level, occupation, income status, as well as the child’s asthma symptoms and a detailed medical history.
- Anthropometric Variables: Each participant’s height, weight, waist circumference, and blood pressure will be measured twice, using certified instruments to ensure accuracy. Height and weight measurements will be carried out using fully calibrated, certified electronic devices. The BMI will be calculated by dividing the weight in kilograms by the square of the height in meters. To measure the waist circumference, a measuring tape will be used at the level of the navel.
- Pulmonary Function Tests: The MasterScreen series pulmonary function testing system from Jaeger (Germany) will be used to conduct pulmonary function tests. Key indicators include the Forced Expiratory Volume in 1 second (FEV_1_), Forced Vital Capacity (FVC), the FEV_1_/FVC ratio, Peak Expiratory Flow (PEF), and the forced expiratory flow at 25%, 50%, and 75% of FVC exhaled (FEF_25_%, FEF_50_%, FEF_75_%) (30).

**Table 1:** The schedule of enrolment, interventions, and assessments.

Study period	Recruitment	Intervention	
Timepoint	0	Discharge	3 Months
Eligibility screen	✓		
Informed consent	✓		
Allocation	✓		
Sociodemographic variables	✓		
C-ACT	✓	✓	✓
PFT	✓	✓	✓
s-EMBU-C	✓	✓	✓
PHQ4	✓	✓	✓
PSSS-SF	✓	✓	✓
NGSES-SF	✓	✓	✓
EQ-5D-5L	✓		✓
Smoking	✓		✓
Alcohol consumption	✓		✓
B-PSQI	✓		✓
FCS-SF	✓		
eHEALS	✓		

B-PSQI, The new Brief Version of the Pittsburgh Sleep Quality Index-Short Form; C-ACT, Childhood Asthma Control Test; eHEALS, The eHealth Literacy Scale; FCS-SF, The Family Communication Scale-Short Form; EQ-5D-5L, EuroQol-5 Dimensions 5 Levels; PFT, Pulmonary Function Tests; PHQ-4, the abbreviated Patient Health Questionnaire-4; PSSS-SF, The Perceived Social Support Scale; NGSES-SF, The New General Self-Efficacy Scale-Short Form; s-EMBU-C, Short-Form Egna Minnen av Barndoms Uppfostran for Chinese.

Post-discharge sustained engagement will be maintained through WeChat, telephone, or SMS communication to ensure participants remain fully informed and actively involved. During these interactions, participants will receive timely updates on research progress, resolution of any emerging concerns, and prompt reminders for upcoming follow-up assessments.

Standardized data entry for this study will be performed using an electronic data capture (EDC) system. First, double data entry by two independent operators will ensure accuracy, followed by logical validation checks on all raw data. Sensitive information will be encrypted using the Unicode encoding scheme. Data will be stored on password-protected servers with role-based access control (only authorized researchers may access de-identified data), and a real-time backup system will be deployed to off-site cloud storage to ensure data integrity and disaster recovery capabilities.

After collecting the relevant data of the participants, we will immediately take measures to delete all personally identifiable information, so as to protect the confidentiality of the data. This process involves replacing personally identifiable information with a unique code. Only a few authorized research team members can access the key connecting these codes with the actual identification information. The key will be stored in a separate and highly secure place. Without the explicit and informed consent of the participants, we will not share any personal information with third parties under any circumstances. All members of the research team are obliged to sign a confidentiality agreement. These agreements are designed to ensure that each team member complies with the highest standards of privacy. From the beginning to the end or even after the end of the study, all personal information will be securely stored on a dedicated server. Only authorized researchers can access the server.

### 2.11 Questionnaires

1. Childhood Asthma Control Test (C-ACT): The C-ACT is an important tool for measuring how well asthma symptoms are controlled in children. It has two parts with a total of seven questions in Chinese version. The first part has four questions for the child to answer. Each question is scored from 0 to 3, based on how the child feels about their asthma control. The second part has three questions for the parent to answer. Each question is scored from 0 to 5, based on how often the child has asthma symptoms in the last four weeks. The total score of the C-ACT goes from 0 to 27. A score of 23 or higher means asthma is fully controlled, with few symptoms affecting daily life. A score between 20 and 22 means partial control, where symptoms are somewhat managed, but some problems remain. A score of 19 or less means asthma is not controlled, and stronger treatments may be needed (31).
2. Short-Form Egna Minnen av Barndoms Uppfostran for Chinese (s-EMBU-C): The s-EMBU-C is a questionnaire designed to evaluate how children see their parents’ parenting styles. The child fills out this questionnaire. There are separate versions for fathers and mothers. Each version has 21 questions, divided into three parts: rejection (6 items), emotional warmth (7 items), and overprotection (8 items). The questions are scored using a scale from 1 to 4. “Never” gets 1 point, “Occasionally” gets 2 points, “Frequently” gets 3 points, and “Always” gets 4 points. Item 15 is scored in reverse. The scores for each part are found by adding up the points for each question in that part. A higher score in a part means the parent shows that parenting style more often. The total score for both versions can range from 28 to 112 points (32).
3. The assessment of depression and anxiety will use the Chinese version of the Patient Health Questionnaire-4 (PHQ-4). This is a short self-report tool to check depression and anxiety symptoms over the past two weeks. The PHQ-4 has four questions. The first two questions focus on depression symptoms, and the last two focus on anxiety symptoms. Each question is rated on a scale from 0 to 3: “Not at all” (0 points), “Several days” (1 point), “More than half the days” (2 points), and “Nearly every day” (3 points). The total score goes from 0 to 12. In studies in China, the PHQ-4 has shown high validity and reliability, with a Cronbach’s alpha of 0.833 (33).
4. The Chinese version of the Perceived Social Support Scale-Short Form (PSSS-SF) is used to measure the level of social support a person feels they receive. The scale has three questions, each about a different type of support: family support (Item 1), friend support (Item 2), and other support (Item 3). Each question is scored on a 7-point scale from 1 (Strongly Disagree) to 7 (Strongly Agree). The total score can range from 3 to 21. Higher scores mean more social support, showing that the person’s social network provides more help, both emotionally and practically (34).
5. The Chinese version of the New General Self-Efficacy Scale (NGSES-SF) is used to measure a person’s confidence in their ability to succeed in tasks or face challenges. It has three questions, each focusing on a different aspect of self-efficacy. Item 1 looks at how capable a person feels in handling a situation. Item 2 focuses on how confident a person is in their skills. Item 3 looks at how confidence in one area can apply to other areas. The NGSES-SF uses a 5-point Likert scale. Respondents can choose from 1 (Strongly Disagree), showing low confidence, to 5 (Strongly Agree), showing high confidence. The total score can range from 3 to 15 (35).
6. The Chinese version of the EQ-5D-5L scale (EuroQol-5 Dimensions 5 Levels) will be used to measure quality of life. The scale looks at five important health areas: mobility, self-care, usual activities, pain/discomfort, and anxiety/depression. For each area, there are five levels, ranging from “no problems” to “extreme problems.” This lets respondents choose the level that best matches their condition. It gives a complete view of a person’s health in these five areas. The EQ-5D-5L also includes a Visual Analogue Scale (VAS). This part lets participants rate how they feel about their current health on a scale from 0 to 100. A score closer to 0 means worse health, while a score closer to 100 shows better health (36).
7. Smoking behavior will be assessed using a self-designed questionnaire. The questionnaire has four multiple-choice questions to categorize smoking habits. These questions are meant to find out: whether the person smokes, how long it has been since they quit smoking (if they quit), how many cigarettes they smoke daily, and when they started smoking. The first question is “Do you have a smoking habit?” with these options: (1) Yes, I smoke regular cigarettes; (2) Yes, I smoke e-cigarettes; (3) I smoke both; (4) I used to smoke (now quit); (5) I have never smoked. Based on their answers, participants will be grouped into two categories: smokers, which include people who currently smoke regular cigarettes, e-cigarettes, or both; and non-smokers, which include those who have never smoked and those who have quit smoking (37).
8. Alcohol consumption will be evaluated using a self-developed 7-item multiple-choice questionnaire designed to assess: 1) current/historical alcohol use status, 2) age of drinking initiation, 3) age of drinking cessation (if applicable), 4) preferred alcoholic beverage types, 5) average daily alcohol intake, 6) pre-quitting daily consumption volume, and 7) perceived anxiety related to alcohol cessation. The instrument begins with the core screening question: ‘Which best describes your alcohol consumption pattern?’ Respondents choose from four mutually exclusive categories: (1) Never consumed alcohol; (2) Current regular drinker; (3) Former drinker who has quit; (4) New-onset drinker in the past year (43, 44).
9. The Chinese version of the Pittsburgh Sleep Quality Index Short Form (PSQI-SF) is a shorter version of the Pittsburgh Sleep Quality Index, made to measure sleep quality. The scale has six items, covering five key aspects of sleep. Items 1 and 2 measure sleep efficiency. Item 3 measures how long it takes to fall asleep. Item 4 looks at sleep duration. Item 5 checks for sleep disturbances. Item 6 looks at overall sleep quality. The total score on the PSQI-SF goes from 0 to 15. A higher score means worse sleep quality (38).
10. The Chinese version of the Family Communication Scale Short Form (FCS-SF) is made to measure how well family members communicate. The scale has four items that assess family communication. It uses a 5-point Likert scale, where 1 means “strongly disagree” and 5 means “strongly agree.” The total score goes from 4 to 20. A higher score means better communication among family members (39).
11. The Chinese version of the Electronic Health Literacy Scale (eHEALS) measures how well parents of children can search for, evaluate, and use electronic health information to solve health problems. The scale has five items in one dimension. It uses a 5-point Likert scale, where 1 means “strongly disagree” and 5 means “strongly agree.” The total score ranges from 5 to 25. A higher score means better electronic health literacy (40).

### 2.12 Statistical analysis

This study will collect quantitative data through questionnaires and process the data using Microsoft Excel. Data analysis will follow the intention-to-treat principle. For continuous variables, descriptive statistics will show means ± standard deviations or medians and interquartile ranges (P25, P75), depending on the data. Normally distributed continuous variables will be compared using the Student’s t-test or one-way analysis of variance (ANOVA). Non-normally distributed data will be analyzed with non-parametric tests. The normality of the data will be checked using the Kolmogorov-Smirnov test. Categorical variables will be shown as sample percentages (n) and analyzed using the Chi-square test. For missing data, multiple imputation using chained equations (MICE) will be used, with 50 iterations to ensure convergence. The imputation method will be predictive mean matching (PMM), with 5 neighbors and 20 imputed datasets.

In the cross-sectional data analysis, this study will use linear regression, logistic regression, and structural equation modeling (SEM) to examine the relationships between behavioral patterns and mobile health applications. Given the nested structure of parent-child dyads within families, we employed multilevel models with family-level random intercepts to account for within-family correlations. For child health outcomes (asthma-related events and symptoms), a two-level negative binomial model was utilized to estimate IRRs and 95% CIs, adjusting for family-level variables (parents’ parenting styles, household income, marital status) and child-level variables (age, sex). For parental outcomes (continuous data), a two-level linear mixed model was applied to evaluate intervention effects, adjusting for family-level variables (marital status, household income) and parent-level variables (age, education, parents’ parenting styles). *Post hoc* comparisons of estimated marginal means were conducted using the emmeans package to test between-group differences. Model fit was validated through convergence checks, residual analysis, and overdispersion testing for count data. All analyses were performed in R (version 4.3.1; R Foundation for Statistical Computing, Vienna, Austria) using the lme4 package (version 1.1-33). An independent statistician with no conflicts of interest conducted the analysis. All statistical tests were two-tailed, with a significance level of 0.05.

After the intervention, we will conduct structured interviews with participants to evaluate the attractiveness, acceptability, usability, and overall satisfaction with the AI-HEALS-based intervention model. Participants will be encouraged to share their personal experiences using the system, provide specific feedback on features that need improvement, and discuss motivations and challenges related to maintaining effective childhood asthma management behaviors. When choosing participants, we will consider sociodemographic factors such as the child’s gender, place of residence, parental education level, and asthma severity. The interview will cover topics such as personal experiences, suggestions for improving the AI-HEALS system, reasons for maintaining self-management behaviors, and challenges faced. We will keep recruiting participants until we reach data saturation to obtain a wide range of information. All interviews will be recorded and transcribed word-for-word to ensure anonymity, and the data will be analyzed using NVivo 12 (Version 12, QRS International, Doncaster, Australia).

## 3 Study management

The Data Monitoring Committee (DMC) will consist of at least two members from the Ethics Committee of Inner Mongolia Maternal and Child Health Hospital, who have no conflict of interest with the study. The committee will be responsible for conducting regular monitoring and review of the study, reporting the findings to the Ethics Committee to ensure the process is independent from the principal investigators. If the DMC identifies any deviations from the approved study protocol or unauthorized changes during the monitoring process, the committee has the authority to suspend or terminate the study. The hospital’s Ethics Committee will conduct a review every six months to assess progress and ensure adherence to the established protocol. Prior to the start of the trial, the principal investigator will meet with the research team weekly to ensure that all procedures comply with the protocol standards. During the data collection phase of the trial, the research team will hold monthly meetings, and the principal investigator and trial manager will maintain daily communication to ensure protocol adherence.

During the study, the research team must report any Serious Adverse Events (SAEs) or Adverse Events (AEs) to the Ethics Committee quickly. If a participant has any SAEs or AEs, even if it is not directly related to the study, the researchers must report it to the research team within 24 hours and stop the trial. If a serious programming error occurs with the AI system or if significant problems are found in user interactions, the researchers must stop the trial right away. The team will fix the technology and improve the user interface of the AI system. After the problem is fixed and checked again, the study can be redesigned and restarted to ensure the participants are safe and the research is valid.

Before the study starts, experts will organize training sessions and evaluations to ensure all team members understand the procedures and technical details. To reduce the risk of missing follow-up, we will keep a list of all participants and assign each one a unique identification code. Also, to ensure both the intervention and control groups are consistent, all survey times will be the same, and the same validated questionnaires will be used. To make sure the data is accurate, we will use a double data entry and checking process, which will improve data quality. We will also talk to statistical experts regularly to choose the best methods for the study. During data processing, if we find any abnormal data, we will check the original questionnaires to make sure the data is correct. Only after confirming that the data is fully accurate will we use it for the final analysis.

The principal investigators will regularly check the work of the research team to make sure the study protocol is followed and the data is collected properly. If the team does not follow the survey and follow-up procedures, the investigators may take action, such as stopping the trial or changing the randomization plan. After these changes, data collection will start again. If there are problems with the questionnaire design or mistakes in recording data, the team will stop the trial, fix the issues, and redesign the questionnaire. After making the changes, the updated questionnaire will be given to participants to ensure the data matches the study’s goals and expected results. This will help keep the study’s science and data reliable, ensuring the results are valid. The data will be managed by a data administrator. The trial manager will oversee data recording and handle any problems with cloud storage, data management, and access. The trial manager will also check regularly to ensure the data is accurate, reliable, and follows the study protocol.

This study allowed unblinding in special situations. These situations include urgent medical needs, such as when a participant has a serious adverse event or needs urgent treatment where knowing the group assignment is important. Unblinding can also happen for valid medical reasons by the participant or their healthcare provider after the study ends. The unblinding process starts with a written request from the principal investigator or a member of the clinical oversight team. The Clinical Review Panel will review all unblinding requests to decide if the conditions are met. If approved, the study team will securely reveal the group assignments following a set protocol.

All proposed changes to the study will first be discussed by the steering committee. After agreeing, the changes will be sent to the hospital’s ethics committee and the research department for approval. Once approved, an official document with the changes will be given to all relevant parties, and an electronic copy will be available if needed. Any changes from the protocol will be recorded by the trial leader and signed by the principal investigator. This document will be kept with the other trial records. After getting approval from the steering committee, research department, and ethics committee, the trial leader will update the clinical trial application.

## 4 Dissemination plans

The research team will compile a summary of the RCT results and send it via email to participants who have indicated an interest in the results on the consent form. The findings will be disseminated through publication in peer-reviewed journals and presentations at both national and international conferences. Additionally, the results will be made available to other hospitals upon request, for sharing with their medical staff. Data will also be provided upon reasonable request (45, 46).

## 5 Discussion

Childhood asthma is a chronic inflammatory airway disease characterized by recurrent wheezing, shortness of breath, and coughing (1, 2). According to global health statistics, asthma remains one of the most common chronic diseases among children, significantly affecting their quality of life, school attendance, and overall health (1, 2). Traditionally, the treatment of childhood asthma relies on a combination of pharmacological therapies and behavioral interventions (6, 7). However, adherence to prescribed treatments and lifestyle modifications remain a challenge (6, 7). Therefore, there is an urgent need to explore innovative approaches to improve asthma control in children, and the emergence of large language models offers promising opportunities to address this need.

The AI-HEALS system is designed to leverage AI technology and mobile health to enhance asthma management, provide personalized health education, and assist behavior change for children with asthma and their families. This system offers knowledge-based question-and-answer services, monitoring of health indicators, education, and support to address the unique needs of pediatric asthma patients and their families. Additionally, AI-HEALS enables healthcare providers to monitor real-time interactions between families and the system through a backend platform, allowing them to assess health education needs and track participation in intervention plans, thereby ensuring the effectiveness of the intervention. Early studies suggest that mobile health technologies have significant potential to improve health outcomes in asthma management (41, 42). Specifically, this system, with the support of large language model technology, holds promise for further improving symptom control in children with asthma.

Based on this, the study will conduct a pilot randomized controlled trial to assess the effectiveness and acceptability of the AI-HEALS system in controlling asthma in children. The main purpose was to evaluate whether the intervention of C-ACT measurement after the intervention improved the control of asthma symptoms. Secondary outcomes include physical health indicators such as lung function, BMI, and hospitalization rates, as well as behavioral and psychological outcomes related to parental involvement, self-management strategies, and overall child quality of life. These outcomes will be measured using quantitative methods (e.g., standardized questionnaires and physiological assessments) and qualitative interviews. Given the medical and behavioral factors involved in childhood asthma, AI-HEALS has great potential to increase patient engagement and improve adherence to treatment regimens.

Although the interventions proposed in this study show considerable potential, some limitations must be recognized. First, the generalizability of the findings may be limited by inclusion criteria that focus on specific populations and clinical characteristics. For example, this study focused on children with asthma who were willing to use digital health interventions and may not have included those with lower health literacy or those less comfortable using technology. Second, while this study will provide valuable insights into the efficacy of the AI-HEALS system, there may be challenges in interpreting the numerous external factors that influence asthma control. Environmental triggers, socioeconomic status, and access to healthcare are all important variables that may influence outcomes, but it may be difficult to fully control for them in the real world. This study will include demographic and clinical covariates in its analysis in an attempt to account for some of these factors, although residual confounders may still affect outcomes. Finally, although the study aims to ensure data accuracy through rigorous monitoring procedures, issues such as participant dropout or missing data may still affect the validity of the findings. The research team plans to use multiple interpolation techniques to address these challenges, but the potential bias of missing data must still be considered.
